# NEGATIVE AGING STEREOTYPES: FEAR OF DEPENDENCY EFFECTS ON ANXIETY AND DEPRESSION IN MIDDLE AND OLDER ADULTS

**DOI:** 10.1093/geroni/igad104.1210

**Published:** 2023-12-21

**Authors:** Darby Mackenstadt, Carolyn Adams-Price

**Affiliations:** Mississippi State University, Mississippi State, Mississippi, United States; Mississippi State University, Mississippi State, Mississippi, United States

## Abstract

Negative stereotypes of aging tend to depict older adults as frail or a burden on society. According to Stereotype Embodiment Theory, adults who believe negative aging stereotypes tend to internalize these thoughts, and have been found to experience negative physical and mental outcomes, in later life. One aspect of these internalized stereotypes is the fear of being dependent on other people, which highlights the older adults’ worry of being seen as incompetent or child-like. Participants were aged 50 to 82-years-old (M=58.5), and primarily White (87%) women (62%). This study explored the relationship between Fear of Dependency Scale (FoD) and anxiety and depression, evaluating gender, age, and physical health as moderators. FoD was related to higher ratings of depression and anxiety in women, with age and physical health acting as moderators. When reporting a high fear of dependency, middle-aged women and women reporting poor physical health had the highest reported anxiety and depression. The early impact of FoD on women is intriguing. FoD was positively associated with higher ratings of depression for men but not anxiety, with only physical health acting as a moderator. Men with high FoD scores and poor physical health reported the highest levels of depression. It appears the negative views of aging can affect individuals, even before they reach older adulthood, and especially if they have poor perceptions of their physical health. Having negative perceptions of aging can negatively impact individuals as they age, and some groups may be more at risk for these outcomes than others.

